# Supplementary material for: Health literacy and preventive behaviors toward microplastic contamination among communities in the major river basins of northeastern Thailand
Source: PLoS One. 2026 Mar 6;21(3):e0344784. doi: 10.1371/journal.pone.0344784 (PMC12965693; doi:10.1371/journal.pone.0344784)
Supplement: Attachment — Submitted filename: 21022026 comments.pdf [file pone.0344784.s002.pdf]

PONE-D-25-57984R1

Health literacy and preventive behaviors toward microplastic contamination among communities in the major river basins of northeastern Thailand

Dear Editor,

Thank you for the opportunity to revise our manuscript. We would like to express our gratitude and thank you and the reviewers for the time and effort in providing us with detailed comments and suggestions. Our corrections have been incorporated into the revised manuscript with red highlighted. Please see our response below.

Journal Requirements:

Please review your reference list to ensure that it is complete and correct. If you have cited papers that have been retracted, please include the rationale for doing so in the manuscript text, or remove these references and replace them with relevant current references. Any changes to the reference list should be mentioned in the rebuttal letter that accompanies your revised manuscript. If you need to cite a retracted article, indicate the article's retracted status in the References list and also include a citation and full reference for the retraction notice.

Response: We have thoroughly reviewed the reference list to ensure accuracy and completeness. All cited articles were screened against recognized retraction databases (Retraction Watch, PubMed, and Crossref), and none were found to be retracted. In addition, several references have been updated to incorporate recent high-impact publications (2023–2024), and corresponding citation numbering has been revised throughout the manuscript.

Reviewers' comments:

Reviewer 2

Comments to the Author

1. If the authors have adequately addressed your comments raised in a previous round of review and you feel that this manuscript is now acceptable for publication, you may indicate that here to bypass the "Comments to the Author" section, enter your conflict of interest statement in the "Confidential to Editor" section, and submit your "Accept" recommendation.

Reviewer #2: (No Response)

2. Is the manuscript technically sound, and do the data support the conclusions?

The manuscript must describe a technically sound piece of scientific research with data that

supports the conclusions. Experiments must have been conducted rigorously, with appropriate controls, replication, and sample sizes. The conclusions must be drawn appropriately based on the data presented.
Reviewer #2: Yes

3. Has the statistical analysis been performed appropriately and rigorously?
Reviewer #2: Yes

4. Have the authors made all data underlying the findings in their manuscript fully available?
The PLOS Data policy requires authors to make all data underlying the findings described in their manuscript fully available without restriction, with rare exception (please refer to the Data Availability Statement in the manuscript PDF file). The data should be provided as part of the manuscript or its supporting information, or deposited to a public repository. For example, in addition to summary statistics, the data points behind means, medians and variance measures should be available. If there are restrictions on publicly sharing data—e.g. participant privacy or use of data from a third party—those must be specified.
Reviewer #2: Yes

5. Is the manuscript presented in an intelligible fashion and written in standard English?
PLOS ONE does not copyedit accepted manuscripts, so the language in submitted articles must be clear, correct, and unambiguous. Any typographical or grammatical errors should be corrected at revision, so please note any specific errors here.
Reviewer #2: Yes

6. Review Comments to the Author
Reviewer #2: The authors have responded carefully and appropriately to the comments raised in the previous round of review. The revisions have substantially improved the clarity of the methodology, the transparency of the statistical analyses, and the alignment between the results and the conclusions.

The study design is appropriate for the research objectives, and the data collection procedures and sample size are adequate. The statistical analyses are suitable for the type of data and are conducted with sufficient rigor. Effect estimates and measures of uncertainty are clearly reported, and the interpretation of findings is consistent with the cross-sectional nature of the

study. Importantly, the authors have avoided causal overinterpretation and have clearly acknowledged the key limitations of the study.

The manuscript is generally well written and logically structured. The presentation of results is clear, and tables and figures are informative. A small number of minor language and editorial issues remain, particularly in the Discussion section, where conciseness and consistency of terminology could be further improved. These issues are minor and do not affect the validity or overall contribution of the study.
Response: We appreciate the reviewer's helpful comments. The Discussion section has been edited to improve conciseness and reduce minor repetition. In addition, terminology has been standardized throughout the manuscript to ensure consistency and clarity. These refinements have further enhanced the overall presentation of the study.

I did not identify any concerns related to research ethics, conflicts of interest, dual publication, or publication ethics. Ethical approval and informed consent procedures are adequately described, and the data availability statement is consistent with journal policy.

Overall, the manuscript is technically sound and has been revised to a standard that is appropriate for publication. Only minor editorial refinements are recommended before final acceptance.
